# Narrative Coherence of Turning Point Memories: Associations With Psychological Well-Being, Identity Functioning, and Personality Disorder Symptoms

**DOI:** 10.3389/fpsyg.2021.623903

**Published:** 2021-03-04

**Authors:** Elien Vanderveren, Annabel Bogaerts, Laurence Claes, Koen Luyckx, Dirk Hermans

**Affiliations:** ^1^Department of Psychology, Faculty of Psychology and Educational Sciences, KU Leuven, Leuven, Belgium; ^2^Faculty of Medicine and Health Sciences, University of Antwerp, Antwerp, Belgium; ^3^UNIBS, University of the Free State, Bloemfontein, South Africa

**Keywords:** narrative coherence, turning point memories, well-being, identity, personality disorder symptoms

## Abstract

Individuals develop a narrative identity through constructing and internalizing an evolving life story composed of significant autobiographical memories. The ability to narrate these memories in a coherent manner has been related to well-being, identity functioning, and personality pathology. Previous studies have particularly focused on coherence of life story narratives, overlooking coherence of single event memories that make up the life story. The present study addressed this gap by examining associations between narrative coherence of single turning point memories and psychological well-being, identity functioning, and personality disorder (PD) symptoms among 333 Belgian emerging adults (72.1% female; *M*_age_ = 22.56, *SD* = 3.13, age range = 18–30). In addition, the present study tested whether narrative coherence could predict unique variance in PD symptoms above and beyond identity and interpersonal functioning, both considered key components of personality pathology. The findings showed that narrative coherence was not significantly related to psychological well-being, but yielded significant negative associations with disturbed identity functioning and antisocial PD symptoms. Furthermore, narrative coherence predicted unique variance in antisocial PD symptoms above and beyond identity functioning, but did not predict unique variance in borderline and antisocial PD symptoms above and beyond both identity and interpersonal functioning. Collectively, these findings suggest that narrative incoherence within single event memories might be characteristic for disturbed identity functioning and antisocial personality pathology.

## Introduction

Identity formation is a fundamental psychosocial task that is characteristic of adolescence and emerging adulthood (Erikson, 1968; Arnett, 2006). The period of emerging adulthood covers the transition from adolescence to adulthood and entails a wide array of identity-related choices in various life domains (Schwartz et al., 2013). According to narrative identity theory, individuals construct an identity by incorporating significant autobiographical memories into an internalized and evolving life story (McAdams, 1992, 2001). More so, research has demonstrated that the ability to construct a *coherent* account of these personal experiences is beneficial for psychological well-being (e.g., Reese et al., 2011) and may contribute to an individual’s sense of unity, purpose, and meaning in life (Habermas and de Silveira, 2008; Vanden Poel and Hermans, 2019). Relatedly, narrative *incoherence* has been linked to borderline personality disorder (BPD; Adler et al., 2012; Jørgensen et al., 2012; Lind et al., 2019b), a clinical disorder marked by severe identity disturbance (American Psychiatric Association [APA], 2013). Along these lines, narrative incoherence might be a deficit in other personality disorders (PDs) as well, as consensus has emerged and research has confirmed that identity disturbance is characteristic of all PDs (American Psychiatric Association [APA], 2013; see Widiger et al., 2018 for a review).

In an attempt to address these theoretical claims and amplify the line of empirical research on this topic, the present study examined associations between narrative coherence within single turning point memories (experiences that have changed the individual’s life or the kind of person he or she is; Mitchell et al., 2020) and psychological well-being, identity functioning, and symptoms of BPD and antisocial personal disorder (ASPD). Additionally, to pinpoint narrative incoherence as a central deficit in personality pathology, this study investigated the incremental validity of narrative coherence in assessing BPD and ASPD symptoms above and beyond identity and interpersonal functioning, both considered key components of PDs (American Psychiatric Association [APA], 2013).

Whereas previous research particularly examined coherence of life story narratives (in its entirety), the present study focuses on coherence of single event memories that make up the life story. *Life story coherence* entails the following three dimensions: the orientation of events in time (temporal coherence), insight into how the events shaped the individual’s life (causal-motivational coherence), and identification of dominant motives and themes underlying various personal experiences (thematic coherence; Habermas and Bluck, 2000). Akin to life story coherence, *narrative coherence of single event memories* can be considered a multidimensional construct. According to Reese et al. (2011, 2014), narratives of single event memories can be considered coherent (1) if the events are situated in time and place, (2) if the events are described in a logical and chronological manner, (3) if the narrative integrates factual information with emotional elaborations and ends with a resolution, and, finally, (4) if it contains explicit references to how the event has shaped the individual’s life or sense of identity. These criteria refer to four distinct dimensions of coherence: context, chronology, theme, and causality.

Narrative coherence has been proposed to represent the primary structural element of narratives in relation to psychological functioning (Adler et al., 2016). Research, albeit limited, has indicated that a higher level of life story coherence relates to more satisfaction with life, less depressive symptoms, and less psychiatric problems (Baerger and McAdams, 1999; Adler et al., 2007; Rasmussen et al., 2017; see Adler et al., 2016 for a review). Similarly, coherence of single event memories has been positively related to psychological well-being (Waters and Fivush, 2015; Reese et al., 2017; Mitchell et al., 2020). Moreover, the ability to construct coherent narratives appears to contribute to psychological resilience, as it has been shown to buffer the psychological impact of negative life events (Vanderveren et al., 2020b). In addition, there are indications that more narrative coherence within single event memories is associated with less stress and less depressive and anxiety-related symptoms in both adolescent (Mitchell et al., 2020) and adult samples (Vanderveren et al., 2019, 2020a; Vanaken and Hermans, 2020).

Importantly, the association between narrative coherence and psychological well-being might be dependent on both narrative valence and gender. Narratives of negative life experiences are typically more coherent as compared to narratives of positive events (Fivush et al., 2003, 2008; Vanderveren et al., 2019, 2020a,b). Additionally, it appears that especially the extent to which individuals are able to construct coherent narratives about *negative* life events relates to psychological well-being (Vanderveren et al., 2019, 2020a). With regard to gender, men appear to be less coherent than women (Thorne and McLean, 2003; Vanderveren et al., 2019, 2020a,b; Vanaken and Hermans, 2020), yet studies suggest that associations between narrative coherence and psychological well-being are stronger in men (Vanden Poel and Hermans, 2019; Vanderveren et al., 2020a).

Furthermore, there is an elaborate theoretical basis regarding the relation between narrative coherence and identity functioning (Waters and Fivush, 2015). Habermas and de Silveira (2008) articulated the importance of being able to construct a coherent life story for healthy identity formation. They assumed that, in addition to achieving coherence within single event memories, individuals must connect and integrate these single coherent narratives into a meaningful and coherent life story as only then it will create a sense of identity. In other words, scholars propose that not merely the content of narratives, but also the *coherence* of narratives might be indicative of identity functioning.

Studies focusing on the life story have indicated that life story coherence is positively related to ego resilience (Pals, 2006), ego development (Mclean and Fournier, 2008), and identity integration (Yampolsky et al., 2013). Research on single event memories has demonstrated that narrative coherence only significantly relates to psychological well-being when narratives include identity-relevant information (e.g., writing about how the event impacted one’s self-definition or personal goals; Waters and Fivush, 2015). However, in this latter study, identity-relevant information was not assessed by means of an independent identity measure, but was only inherently included in the narrative (Waters and Fivush, 2015).

Building on these results, Vanden Poel and Hermans (2019) examined the relation between narrative coherence of single event memories and identity using an independent measure for identity that considers both adaptive and disturbed identity functioning. They reported that narrative coherence is positively associated with consolidated identity functioning, which refers to feeling integrated and whole, experiencing a high degree of self-continuity, and feeling confident about who you are (Kaufman et al., 2015). Alternatively, they found that coherence is negatively associated with disturbed identity functioning and lack of identity (Vanden Poel and Hermans, 2019). Disturbed identity functioning captures normative periods of identity-related confusion and more severe identity difficulties causing distress or developmental delay, whereas lack of identity indicates feelings of non-existence, inner emptiness, and being broken (Kaufman et al., 2015; Kaufman and Crowell, 2018). Moreover, the authors found that these associations are more prominent in men as compared to women (Vanden Poel and Hermans, 2019). Although it is a strongly held assumption within the narrative identity literature that being able to construct coherent narratives is instrumental for healthy identity functioning, systematic research linking narrative coherence to independent measures of identity is lacking.

More recently, as identity and narrative coherence are believed to be linked and identity disturbance is considered a defining characteristic of BPD (American Psychiatric Association [APA], 2013), researchers have also come to study associations between narrative coherence and (symptoms of) BPD. In line with assumptions, individuals with BPD (symptoms) appear to construct less coherent life story narratives as compared to healthy controls (Adler et al., 2012; Jørgensen et al., 2012) or individuals with other clinical diagnoses (such as an eating disorder or an obsessive-compulsive disorder; Rasmussen et al., 2017). Authors assume that specific BPD characteristics such as identity disturbance, interpersonal difficulties, and compromised mentalization might be related to the inability to create a coherent life story (Bateman and Fonagy, 2004; Jørgensen et al., 2012; Lind et al., 2020). However, consensus has emerged that identity disturbance and interpersonal difficulties might be markers of *all* PDs and may not be merely characteristic of BPD (Morey et al., 2011; American Psychiatric Association [APA], 2013). Although research examining narrative coherence in relation to personality pathology has so far exclusively focused on life story coherence and on BPD, narrative incoherence (within single event memories) might be a deficit of other PDs as well.

More so, narrative coherence might be uniquely linked to PD symptoms above and beyond identity disturbance. Although narrative identity has long been considered a primary domain of personality (McAdams, 1995), it is still unclear whether narrative *coherence* is a central feature of personality as well. In support of this last assumption, a recent study demonstrated that self-reported identity disturbance and narrative incoherence appear to capture distinct aspects of BPD (Lind et al., 2019b). Considering this, studies that incorporate multiple assessment methods for identity functioning when assessing PDs may provide a comprehensive understanding of identity disturbance in personality pathology.

Recently, however, studies have shown that identity disturbance and interpersonal problems are strongly intertwined and should both be considered when assessing PDs (Anderson and Sellbom, 2018; Cruitt et al., 2019). Taking interpersonal functioning into consideration when studying associations between narrative coherence and PD symptoms is important as narrative coherence is negatively related to interpersonal difficulties, such as attachment insecurity and poor mentalization (Lind et al., 2020). To date, research studying associations between narrative coherence and PD symptoms considering the roles of both identity and interpersonal functioning are virtually absent.

### The Present Study

The present study addressed three research objectives. First, we examined whether narrative coherence of single turning point memories was significantly associated with psychological well-being, identity functioning, and symptoms of BPD and ASPD. We hypothesized coherence to be positively associated with psychological well-being (Waters and Fivush, 2015; Mitchell et al., 2020) and consolidated identity functioning (Vanden Poel and Hermans, 2019), and negatively with disturbed identity functioning, lack of identity (Vanden Poel and Hermans, 2019), and symptoms of BPD (Adler et al., 2012; Jørgensen et al., 2012; Rasmussen et al., 2017). Furthermore, despite a lack of empirical evidence, we hypothesized narrative coherence to be negatively associated with symptoms of ASPD as well, considering that identity disturbance is an area of dysfunction in all PDs (American Psychiatric Association [APA], 2013; Widiger et al., 2018) and may interfere with the ability to create a coherent narrative (Bateman and Fonagy, 2004; Jørgensen et al., 2012; Lind et al., 2020).

Second, we studied whether narrative coherence could predict unique variance in BPD and ASPD symptoms above and beyond identity functioning. As previous research indicated that self-reported identity disturbance and narrative incoherence capture unique aspects of BPD (Lind et al., 2019b), we hypothesized narrative coherence to predict unique variance in BPD and ASPD symptoms above and beyond self-reported identity functioning.

Third, we tested whether narrative coherence could predict unique variance in BPD and ASPD symptoms above and beyond identity and interpersonal functioning. As our study is the first one to investigate the incremental validity of narrative coherence in assessing PD symptoms above and beyond both identity and interpersonal functioning, we could not formulate specific hypotheses. For the second and third research aim, narrative valence and gender were taken into account. As previously described, studies have demonstrated that narrative coherence and its relation to psychological well-being and identity functioning is dependent upon these two variables (Vanden Poel and Hermans, 2019; Vanderveren et al., 2020a). Relatedly, the associations between narrative coherence and PD symptomatology may be dependent upon narrative valence and gender as well.

## Materials and Methods

### Participants

A total of 335 Belgian emerging adults completed the online survey. Of this initial sample, two participants did not provide a turning point narrative and were consequently excluded for analyses. The final sample comprised 333 participants of which 93 were male (27.9%) and 240 female (72.1%). Participant’s age ranged between 18 and 30 years old (*M* = 22.56; *SD* = 3.13).

### Procedure

The online survey, which was programmed using Qualtrics, was distributed through various social media platforms. After informed consent was obtained, participants were asked to provide one turning point narrative and to complete a series of questionnaires. To reimburse participants for their effort and time, one gift voucher to the value of €50 was randomly distributed per 25 participants. This study was approved by the Social and Societal Ethics Committee of the KU Leuven (G-2020-1966) and was preregistered on AsPredicted^1^.

### Measures

#### Narrative Coherence

Participants were asked to retrieve and write down one significant personal experience that has changed their life or the kind of person they are (i.e., a turning point memory). They were instructed to elaborate on both the facts and the personal meaning of this turning point event. These narratives were later coded for their level of coherence using the *Narrative Coherence Coding Scheme* (Reese et al., 2011, 2014). Scores on each individual dimension (i.e., context, chronology, theme, and causality) were summed up into one overall coherence score. In addition, the valence of the narrative was coded. Previous studies have demonstrated the validity and reliability of this coding system across various age groups (e.g., Reese et al., 2011; Waters and Fivush, 2015). Inter-rater reliability was evaluated by calculating Cohen’s kappa between two independent coders on 10% of the narratives. Reliability analysis indicated excellent reliability across the four dimensions of narrative coherence (κ = 0.94 for context, κ = 0.95 for chronology, κ = 0.90 for theme, and κ = 0.91 for causality). In addition, a Cohen’s kappa of 0.86 was obtained for narrative valence. The remaining narratives were then coded by the corresponding author.

#### Psychological Well-Being

The Dutch translation of the 54-item version of the *Psychological Well-Being Scale* (PWBS; Ryff and Keyes, 1995; Van Dierendonck and Smith, 2001) was conducted to assess participants’ well-being. The PWBS is a self-report questionnaire that measures various categories of psychological well-being (i.e., autonomy, environmental mastery, personal growth, purpose in life, self-acceptance, and positive relations). The items are scored using a six-point rating scale ranging from “strongly disagree” to “strongly agree.” Previous research by Ryff and Keyes (1995) has demonstrated the PWBS to be a valid and reliable instrument. In this study, the internal consistency of the PWBS was 0.95.

#### Identity Functioning

Participants completed the Dutch version of the *Self-Concept and Identity Measure* (SCIM; Kaufman et al., 2015; Dutch translation: Bogaerts et al., 2018). This 27-item self-report questionnaire comprises three subscales: consolidated identity, disturbed identity, and lack of identity. The applicability of each statement is rated on a seven-point Likert scale ranging from “completely disagree” to “completely agree.” The SCIM has been proven to be a valid and reliable measure of identity functioning (Kaufman et al., 2015; Bogaerts et al., 2018). In the present study, the internal consistency was 0.73 for consolidated identity, 0.83 for disturbed identity, and 0.92 for lack of identity.

#### Symptoms of BPD and ASPD

Symptoms of BPD and ASPD were assessed by administering the two corresponding subscales of the *Assessment of DSM-IV Personality Disorders* questionnaire (ADP-IV; Schotte et al., 1998). Participants are asked to rate the typicality of ten borderline and eight antisocial traits on a seven-point Likert scale ranging from “totally disagree” to “totally agree,” yielding a trait-score. When a trait-score exceeds a score of five, participants are asked to indicate to what extent that trait causes them or others distress on a three-point Likert scale ranging from “totally not” to “most certainly,” yielding a distress-score. The ADP-IV produces both dimensional and categorical PD scores. As we conducted our study in a community sample of emerging adults, only dimensional trait-scores were used as an indication of PD symptomatology. Dimensional BPD and ASPD scores were calculated from summing up the trait-scores for each specific PD. The ADP-IV has been established as a reliable and valid measure of personality disorder symptomatology (Schotte et al., 1998; De Doncker et al., 2000). In the present study, the internal consistency was 0.87 for symptoms of BPD and 0.76 for symptoms of ASPD.

#### Interpersonal Functioning

Impairment in self- and interpersonal functioning was assessed using the *Level of Personality Functioning Scale-Brief Form 2.0* (LPFS-BF 2.0; Bach and Hutsebaut, 2018). Participants are asked to rate the applicability of 12 items on a four-point scale ranging from “very false” to “very true.” The psychometric quality of the scale has been established by previous research (Bach and Hutsebaut, 2018). In the present study, the internal consistencies of the self- and interpersonal functioning scales were 0.84 and 0.67, respectively. Only the latter scale was of interest for the present study.

### Data-Analysis

SPSS 27.0 was used to analyze the assembled data. As preliminary analyses, independent-samples *T*-tests were conducted to examine (1) potential differences between positively and negatively valenced narratives with regard to narrative coherence, and (2) gender differences in all variables. Cohen’s *d* was used as a measure of effect size with values of 0.20, 0.50, and 0.80 indicating, respectively, small, medium, and large effects (Cohen, 1988). For the primary analyses, we calculated Pearson and partial correlation coefficients to examine the associations between narrative coherence of single turning point memories and psychological well-being, identity functioning, and symptoms of BPD and ASPD after controlling for narrative valence. A hierarchical linear regression analysis was conducted to investigate narrative coherence as a predictor of PD symptoms above and beyond identity functioning. Finally, a hierarchical regression analysis was conducted to examine the predictive power of narrative coherence with regard to PD symptoms above and beyond identity and interpersonal functioning. Both regression models controlled for gender and narrative valence.

## Results

### Descriptives

Mean and standard deviation of all variables are presented in Table 1. Independent-samples *T*-tests revealed that men reported more overall psychological well-being and more ASPD symptoms, whereas women scored higher on symptoms of lack of identity and BPD (see Table 1). No additional gender differences were observed. The level of coherence was dependent on the valence of the narratives. Independent-samples *T*-tests indicated that negative narratives were more coherent than positive narratives, *t*(329) = 3.26, *p* < 0.01, *d* = 0.38. On a sublevel, negative narratives were more chronological *t*(180.15) = 4.43, *p* < 0.001, *d* = 0.55, and thematically coherent, *t*(183.25) = 3.16, *p* < 0.01, *d* = 0.39. No differences in contextual, *t*(214.97) = 1.58, *p* = 0.12, *d* = 0.18, or causal coherence, *t*(329) = −0.10, *p* = 0.92, *d* = −0.01, were observed.

**TABLE 1:** Descriptive information.

	Total	Males	Females	Gender differences	
Variables	*M (SD)*	*M (SD)*	*M (SD)*	*t*(df)	Cohen’s *d*
Narrative coherence	7.76 (2.72)	7.90 (2.61)	7.70 (2.76)	0.60(331)	0.07
Psychological well-being	4.08 (0.63)	4.20 (0.58)	4.03 (0.64)	2.16*(312)	0.27
Consolidated identity	5.00 (0.73)	4.96 (0.70)	5.02 (0.74)	−0.75(331)	−0.09
Disturbed identity	3.05 (0.88)	2.94 (0.80)	3.09 (0.90)	−1.41(331)	−0.17
Lack of identity	2.92 (1.37)	2.60 (1.14)	3.05 (1.43)	−3.01**(209)^a^	−0.33
BPD symptoms	2.86 (1.21)	2.53 (1.03)	2.99 (1.25)	−3.29**(187.07)	−0.38
ASPD symptoms	1.70 (0.72)	1.85 (0.71)	1.64 (0.71)	2.30*(311)	0.29
Impairment in interpersonal functioning	1.88 (0.52)	1.85 (0.53)	1.89 (0.52)	−0.58(314)	−0.07

*p < 0.05, **p < 0.01. ^a^Independent T-test adjusted for unequal variance across gender. d = 0.2, 0.5, and 0.8 correspond to small, medium, and large effects, respectively (Cohen, 1988).

### Narrative Coherence: Associations With Well-Being, Identity, and PD Symptoms

Pearson correlation coefficients between narrative coherence of single turning point memories and psychological well-being, identity functioning, and symptoms of BPD and ASPD are presented in Table 2 below the diagonal. As the level of coherence was dependent upon the valence of the narratives, the latter was controlled for when investigating associations between narrative coherence and the outcome variables. Partial correlation coefficients, which are presented in Table 2 above the diagonal, resulted in the following observations. First, narrative coherence was not significantly related to psychological well-being. Second, narrative coherence was significantly negatively associated with disturbed identity functioning, but unrelated to consolidated identity functioning or lack of identity. Third, narrative coherence was significantly negatively related to ASPD symptoms, but unrelated to BPD symptoms.

**TABLE 2:** Pearson correlation coefficients between main variables.

	1	2	3	4	5	6	7	8
1 Narrative coherence	–	0.01	–0.02	−0.15**	0.03	–0.00	−0.12*	-0.04
2 Psychological well-being	–0.01	–						
3 Consolidated identity	–0.04	0.59***	–					
4 Disturbed identity	−0.13*	−0.56***	−0.39***	–				
5 Lack of identity	0.06	−0.77***	−0.55***	0.61***	–			
6 BPD symptoms	0.05	−0.69***	−0.49***	0.54***	0.73***	–		
7 ASPD symptoms	–0.09	−0.33***	−0.27***	0.37***	0.32***	0.50***	–	
8 Impairment in interpersonal functioning	–0.04	−0.56***	−0.41***	0.46***	0.49***	0.52***	0.38***	–
9 Age	–0.02	0.14*	0.19***	−0.11*	−0.14**	−0.16**	0.01	-0.10

*p < 0.05, **p < 0.01, *** p < 0.001. Scores above the diagonal represent partial correlation coefficients after controlling for narrative valence.

### Narrative Coherence: A Central Issue in Personality Pathology?

As presented in Table 3, hierarchical linear regression analyses revealed that narrative coherence was a significant negative predictor of ASPD symptoms, though not of BPD symptoms, above and beyond identity functioning. For ASPD symptoms, adding narrative coherence and valence to the regression model resulted in a significant increase in explained variance, *R*^2^_*change*_ = 0.02, *F*(2,304) = 3.22, *p* < 0.05. Finally, as can be seen in Table 4, narrative coherence did not predict BPD and ASPD symptoms above and beyond both identity and interpersonal functioning. Both gender and narrative valence were included in the two aforementioned models as identity functioning and PD symptomatology were dependent on gender and narrative coherence was dependent on narrative valence.

**TABLE 3:** Hierarchical linear regression analysis predicting BPD and ASPD symptoms as a function of gender, identity functioning, and narrative coherence.

	Predictors	Standardizedβ	t	r^2^_*x(y*_._*z)*_
BPD symptoms	Gender	0.09	2.28*	0.01
	Consolidated identity	–0.14	−3.12**	0.01
	Disturbed identity	0.14	2.80**	0.01
	Lack of identity	0.54	9.59***	0.13
	Narrative coherence	0.01	0.22	0.00
	Narrative valence	–0.08	−2.05*	0.01
ASPD symptoms	Gender	–0.17	−3.20**	0.03
	Consolidated identity	–0.09	–1.38	0.01
	Disturbed identity	0.25	3.62***	0.03
	Lack of identity	0.13	1.64	0.01
	Narrative coherence	–0.11	−1.99*	0.01
	Narrative valence	–0.10	–1.85	0.01

*p < 0.05, **p < 0.01, ***p < 0.001.

**TABLE 4:** Hierarchical linear regression analysis predicting BPD and ASPD symptoms as a function of gender, identity functioning, impairment in interpersonal functioning, and narrative coherence.

	Predictors	Standardizedβ	t	r^2^_*x(y*_._*z)*_
BPD symptoms	Gender	0.09	2.35*	0.01
	Consolidated identity	–0.11	−2.38*	0.01
	Disturbed identity	0.09	1.83	0.00
	Lack of identity	0.50	8.94***	0.11
	Impairment in interpersonal functioning	0.18	3.92***	0.02
	Narrative coherence	0.01	0.12	0.00
	Narrative valence	–0.07	–1.82	0.00
ASPD symptoms	Gender	–0.17	−3.24**	0.03
	Consolidated identity	–0.04	–0.66	0.00
	Disturbed identity	0.19	2.78**	0.03
	Lack of identity	0.07	0.95	0.00
	Impairment in interpersonal functioning	0.24	3.87***	0.05
	Narrative coherence	–0.10	–1.81	0.01
	Narrative valence	–0.09	–1.74	0.01

*p < 0.05, **p < 0.01, ***p < 0.001.

## Discussion

The theory of narrative identity posits that the ability to construct a coherent account of personal experiences is beneficial for well-being (Adler et al., 2016) and contributes to an individual’s sense of identity (Habermas and de Silveira, 2008). In addition, narrative incoherence has been linked to BPD (Adler et al., 2012; Jørgensen et al., 2012; Lind et al., 2019b), and might be a deficit in other PDs as well, as consensus has emerged that identity disturbance is characteristic of all PDs (American Psychiatric Association [APA], 2013). Despite theoretical claims, associations between narrative coherence within single event memories and psychological well-being, identity functioning, and PD symptoms are largely unexplored. Accordingly, the present study examined associations between narrative coherence within single turning point memories and psychological well-being, identity functioning, and symptoms of BPD and ASPD. Additionally, to pinpoint narrative incoherence as a central deficit in PDs, the present study investigated the incremental validity of narrative coherence in assessing BPD and ASPD symptoms above and beyond identity and interpersonal functioning.

Regarding the first research objective, the present study was unable to support the hypothesized positive association between narrative coherence and psychological well-being. Although unexpected, other studies have likewise failed to detect a positive association between the two (Vanderveren et al., 2020b) or observed the reverse relation (Waters et al., 2013; Graci et al., 2018). The following two explanations could be relevant in this regard. First, previous studies have operationalized psychological well-being in various ways (e.g., overall well-being, life satisfaction, personal growth, absence of psychological problems, etc.). Vanden Poel and Hermans (2019) proposed the possibility that narrative coherence might be exclusively beneficial for specific components of well-being, potentially explaining the inconsistent findings in the literature. Second, it has been previously suggested that certain characteristics specific to the event narrated upon (e.g., retention interval and emotional intensity) and to the narrator (e.g., ruminative response style), which are usually not accounted for, might affect the association between narrative coherence and well-being (Waters et al., 2013; Fivush et al., 2017; Vanderveren et al., 2020a). So, although narrative coherence is often considered the primary structural element of narratives in relation to psychological well-being (Adler et al., 2016), empirical research seems to suggest their relation to be more complex than originally assumed.

In line with our hypothesis, narrative coherence within single turning point memories was significantly negatively associated with disturbed identity functioning. Individuals who narrate about their personal experiences in a more coherent manner appear to experience less identity-related disturbance such as temporary or more permanent feelings of identity confusion and incoherence (Kaufman et al., 2015). In accordance with theoretical claims (Bluck and Alea, 2002; Habermas and de Silveira, 2008), this finding suggests that narrative incoherence within single turning point memories might be an important characteristic of disturbed identity functioning in emerging adulthood.

Contrary to our expectations based on earlier reports from Vanden Poel and Hermans (2019), narrative coherence within single turning point memories was not significantly related to consolidated identity functioning and lack of identity. These findings suggest that individuals’ overall sense of identity consolidation or a complete lack thereof may not be related with coherence on the level of single event memories. Highlighting identity as a configuration of various elements, consolidated identity captures the feeling of being integrated and whole, whereas lack of identity signals feelings of being broken or fragmented (Kaufman et al., 2015). Relatedly, Erikson (1968) has conceptualized consolidated identity as the extent to which different elements of one’s identity fit together into an integrated whole (Schwartz et al., 2009). Therefore, although merely speculative, individuals’ sense of consolidated identity or lack of identity may rather be associated with the coherence of their life story.

With regard to the associations between narrative coherence and PD symptoms, the present study demonstrated a significant negative association between coherence and ASPD symptoms after controlling for narrative valence. Individuals who constructed more coherent narratives reported significantly less ASPD symptoms. This finding aligns well with research showing a negative association between narrative coherence and behavioral problems in young individuals (such as violent behavior; Maruna, 2001; Hauser et al., 2006; Wainryb et al., 2010; Lind et al., 2020). Relatedly, previous studies indicated a lack of coherence in narratives produced by psychopathic individuals (Eichler, 1965; Brinkley et al., 1999). Researchers assume that the development of a coherent narrative may play a functional role in processes of resilience and desistance from criminal offending (Maruna, 2001; Hauser et al., 2006). Lind et al. (2020) add that poor abilities to construct coherent narratives might reflect a lowered competence to carry out well-structured and thoughtful actions in daily life.

Inconsistent with our expectations, the results did not provide support for the hypothesized negative association between narrative coherence and BPD symptoms. We propose three potential explanations for this null result. First, different from previous studies (Adler et al., 2012; Jørgensen et al., 2012; Rasmussen et al., 2017; Lind et al., 2019a), the present study did not focus on life story coherence in clinical samples, but studied coherence within single event memories among community adults. These methodological differences might contribute to the failure to replicate.

A second explanation for why we obtained a significant negative association between narrative coherence and ASPD but not with BPD symptoms may be found in previous research examining associations between identity functioning and PDs using the Self-Concept and Identity Measure (SCIM; Bogaerts et al., 2018, in press; Kaufman et al., 2015). Across these studies, disturbed identity appeared to be a strong positive predictor of ASPD symptoms, whereas lack of identity appeared to be particularly predictive of BPD symptoms. Considering that narrative coherence was not significantly related to lack of identity in the present study, narrative coherence might be less likely related to BPD symptoms as well.

Third, although speculative, these results may be an indication of the gender differences found in how narrative coherence relates to identity functioning. Vanden Poel and Hermans (2019) demonstrated that the association between narrative coherence and disturbed identity functioning is more prominent in men than in women. Given that ASPD symptoms might be particularly characteristic of males and BPD symptoms particularly characteristic of females (Paris, 2004), it may be more likely to obtain a significant association between narrative coherence and ASPD symptoms. Taken together, although we could not replicate the hypothesized negative associations between narrative coherence and BPD symptoms, our results appear to imply that narrative incoherence might not be uniquely related to BPD (symptoms), but might be a feature of other PDs as well.

Regarding the second research objective, narrative coherence could predict unique variance in ASPD symptoms (though not in BPD symptoms) above and beyond identity functioning. In line with results of Lind et al. (2019b), these findings seem to suggest that narrative coherence and self-reported identity disturbance capture unique and distinct aspects of personality pathology.

Regarding the third research objective, narrative coherence did not predict unique variance in BPD and ASPD symptoms above and beyond both identity and interpersonal functioning. These results suggest that, when considering identity disturbance and interpersonal problems (i.e., key characteristics of PDs), narrative coherence does not significantly contribute to the prediction of BPD and ASPD symptoms. Thus, narrative incoherence may not be a primary dysfunction in personality pathology. Nevertheless, these findings provide further support for the Alternative Model for Personality Disorders (AMPD) forwarded in Section III of DSM-5 (American Psychiatric Association [APA], 2013). In accordance with the AMPD and previous research (see Widiger et al., 2018 for a review), our findings suggest that identity disturbance and interpersonal difficulties are central commonalities across PDs.

The following limitations to the present study should be considered when interpreting the findings. First, narrative coherence was assessed by asking participants to narrate about one single turning point event. To obtain a more comprehensive estimation of someone’s ability to construct coherent narratives, it would be recommended for future studies to use a compound measure of narrative coherence by instructing participants to narrate about various types of single events in addition to a turning point (e.g., high or low point events). Second, the study exclusively consisted of self-report measures, which could result in inflated correlations between studied variables due to shared method variance (Podsakoff et al., 2003). Future studies should therefore include multi-method and multi-informant assessment methods. Third, the cross-sectional design of the present study inhibits us from drawing conclusions regarding the causality and directionality of the observed associations. To overcome this limitation, experimental and longitudinal designs are required. Fourth, our sample was characterized by an overrepresentation of female community adults. Due to the homogeneous nature of our sample, caution is recommended when interpreting the present findings and generalizing the obtained results to other more diverse or clinical samples. Future studies should include an equal ratio of male and female participants and should replicate the findings in a clinical sample. Lastly, it should be noted that the observed associations between narrative coherence, disturbed identity functioning, and ASPD symptomatology are rather weak. This may suggest that narrative coherence may not be as strongly related to identity functioning as commonly hypothesized and/or that other variables might affect the strength of these relations (e.g., retention interval, emotional intensity, centrality of events for the self). Replicating the present findings is therefore recommended.

In addition to remedying the previously mentioned limitations, future research investigating the relative centrality of narrative incoherence across various PDs would be a valuable addition to the present study. Relatedly, as narrative identity is considered to represent a distinct domain of personality (McAdams, 1992), it would be recommended for future studies to identify how narrative incoherence, supposedly the central structural characteristic of narrative identity (Adler et al., 2016), manifests and interacts with other core domains of personality pathology such as identity and interpersonal functioning. Such future studies should explore potential mechanisms that might explain the relation between narrative coherence and both identity functioning and PD symptomatology. In doing so, it would be recommended to include information on other demographic variables to consider the effects of individual and/or contextual factors on narrative coherence and how it relates to identity functioning and PD symptomatology.

To conclude, the present study contributes to the narrative identity literature by investigating the association between narrative coherence of single turning point memories and both identity functioning and PD symptomatology. Results indicate that narrative coherence is negatively associated with disturbed identity functioning and is uniquely related to ASPD symptoms over and above identity functioning (though not over and above identity *and* interpersonal functioning). Although caution is warranted and additional research is required, these results seem to suggest that narrative coherence and self-reported identity disturbance capture unique and distinct aspects of identity functioning in personality pathology.

## Data Availability Statement

The raw data supporting the conclusions of this article will be made available by the authors, without undue reservation.

## Ethics Statement

The studies involving human participants were reviewed and approved by the Social and Societal Ethics Committee of KU Leuven. The patients/participants provided their written informed consent to participate in this study.

## Author Contributions

EV and AB designed the study, collected and analyzed the data, and wrote the first draft of the manuscript. KL, LC, and DH critically revised the manuscript. All authors contributed to the article and approved the submitted version.

## Conflict of Interest

The authors declare that the research was conducted in the absence of any commercial or financial relationships that could be construed as a potential conflict of interest.

## Footnotes

1 https://aspredicted.org/y82uj.pd
